# Right ventricular longitudinal function is linked to left ventricular filling pressure in patients with repaired tetralogy of fallot

**DOI:** 10.1007/s10554-022-02728-3

**Published:** 2022-09-17

**Authors:** Martin Johansson, Edem Binka, Benjamin Barnes, Lasya Gaur, Erik Hedström, Shelby Kutty, Marcus Carlsson

**Affiliations:** 1 Department of Clinical Sciences Lund, Clinical Physiology, Lund University, Skåne University Hospital, Lund, Sweden; 2 Department of Clinical Sciences Lund, Pediatric Anesthesia and Intensive Care, Lund University, Skåne University Hospital, Lund, Sweden; 3 Division of Pediatric Cardiology, Johns Hopkins Hospital, Baltimore, MD USA; 4 Department of Pediatrics, Blalock-Taussig-Thomas Heart Center, Johns Hopkins Hospital, Baltimore, MD USA; 5 Department of Clinical Sciences Lund, Diagnostic Radiology, Lund University, Skåne University Hospital, Lund, Sweden; 6 National Heart, Lung, and Blood Institute, NIH*, Maryland, USA

**Keywords:** Congenital heart disease, Cardiac magnetic resonance imaging, Pulmonary insufficiency, Right ventricular function

## Abstract

Experimental data on pulmonary regurgitation has linked right ventricular longitudinal function to left ventricular filling pressure in animals with induced and treated pulmonary regurgitation but this relationship has not been investigated in patients with repaired Tetralogy of Fallot (rToF). The aim of this study was to determine if right ventricular longitudinal function assessed using cardiovascular magnetic resonance (CMR) is associated with left ventricular filling pressure in patients with rToF. A second objective of this study was to determine if direction of septal movement is related to right ventricular pressure load in rToF. Eighteen patients with rToF undergoing CMR and heart catheterization prior to pulmonary valve replacement were retrospectively included and catheter-based pressure measurements were compared with CMR-derived RV regional function. Left ventricular filling pressure was measured as precapillary wedge pressure (PCWP). Longitudinal contribution to RV stroke volume correlated with PCWP (r = 0.48; p = 0.046) but not with RV EF or pulmonary regurgitation. Neither RV longitudinal strain nor TAPSE showed correlation with PCWP. Longitudinal contribution to stroke volume was lower for the RV compared to the LV (49 vs 54%; p = 0.039). Direction of septal movement did not show a correlation with RV end-systolic pressure. Right ventricular longitudinal pumping is associated with left ventricular filling pressure in rToF-patients and this inter-ventricular coupling may explain LV underfilling in patients with pulmonary regurgitation and rToF and may be of value to determine right ventricular dysfunction. RV systolic pressure, however, cannot be assessed from the direction of septal movement, in these patients.

## Introduction

Tetralogy of Fallot (ToF) is one of the most common complex congenital heart defects. After initial surgical correction early in life many develop progressive right ventricular (RV) systolic dysfunction and RV dilatation [1]. Right ventricular hypertrophy and dysfunction are predictors of death and adverse cardiac events [2].

Depending on type of initial repair and the morphological appearances of the RV outflow tract, pulmonary valve (PV), and pulmonary artery or conduit, ToF might include right ventricular outflow obstruction, pulmonary stenosis (PS), pulmonary regurgitation (PR) or a combination thereof [3]. Pulmonary valve replacement (PVR) improves long-term outcome in repaired ToF (rToF) [3, 4], as it alleviates PR. The main imaging criteria to perform PVR are increasing RV systolic dysfunction and dilatation of the RV. However, the optimal time-point for PVR after primary surgery is still being frequently debated.

The right ventricle in rToF can be exposed to exaggerated pressure load [5], volume load [4] or a combination thereof and can be evaluated using invasive pressure measurements. During catheterization, precapillary wedge pressure can be measured as a proxy of the filling pressure of the left atrium [6], however, this does not always accurately describe preload of the left ventricle [7]. To quantify ventricular volumes and function, CMR is commonly used and allows for determination of regional contributions (within the ventricle) to stroke volume [8, 9]. CMR measurements used to evaluate RV function include the longitudinal component of contraction caused by longitudinal shortening and the movement of the atrioventricular plane towards the apex of the heart, i.e. the atrioventricular plane displacement (AVPD) [10], as well as the radial movement of the walls of the ventricle. Radial movement can in turn be divided into septal and lateral contributions to stroke volume. To put this into context, 60% of left ventricular pumping and 80% of right ventricular pumping in the healthy human heart can be attributable to this longitudinal contraction [11]. However, in patients with rToF and PR longitudinal contribution has been shown to account for 50–60% of the left and only 30% of the right ventricular stroke volume [8]. To further assess ventricular function, the shape and movement of the septum is clinically used to detect increased RV pressure load but it is not known if this is applicable in rToF patients where there is a concurrent volume load as well as restrictive right ventricular physiology. Conduction abnormalities are common in rToF [3], especially right bundle branch block which potentially leads to asynchronous septal contraction. Whether this in turn affects the septal contribution to right ventricular stroke volume has not been determined.

Experimental data on pulmonary regurgitation has linked RV longitudinal function to left ventricular filling pressures [12]. Kopic et al. found a high correlation (R = 0.97 P < 0.001) between the longitudinal contribution to RV stroke volume and invasively measured precapillary wedge pressure (PCWP) in an animal model of pulmonary regurgitation [12], without pulmonary pathology. This indeed spikes interest. However, the size of PR did not correlate with the PCWP, leading to the thought that decreased RV longitudinal function might be used as an independent variable to assess the effect of PR on LV underfilling.

In this study we aimed to elucidate further the relationship between invasive pressure measurements and RV regional function as well as direction of septal movement in a cohort of patients with rToF. We hypothesized that RV longitudinal pumping is related to the filling pressures of the left ventricle in this category of patients and that there is no correlation between direction of septal movement and pressure load of the RV.

## Materials and methods

### Study design

Retrospective inclusion of patients with rToF undergoing cardiac catheterization and CMR imaging prior to scheduled pulmonary valve replacement. Indications for PVR included RV dilatation, clinical symptomology due to stenosis, and invasive pressure indices. This retrospective aspect of the study was approved by the Johns Hopkins Institutional Review Board.

### Catheterization

Right heart catheterization by femoral venous access using a 5–7 French Balloon wedge-pressure catheter (Arrow International, Inc., Morrisville, NC, USA) included measurements of oxygen saturation and pressure. A pressure drop across the pulmonary valve of more than 30 mmHg was classified as pulmonary stenosis. Pulmonary hypertension was defined as a transpulmonary pressure greater than 12 mmHg. Pulmonary capillary wedge pressure was measured in the left pulmonary artery, right pulmonary artery, and if applicable in the conduit from primary surgery.

### Cardiac magnetic resonance imaging

Cardiac magnetic resonance imaging was conducted using a Siemens 1.5 T (Siemens Healthcare, Erlangen, Germany) with retrospective ECG gating at end-expiratory breath hold. Cine imaging was acquired with a steady state free precession sequence with typical repetition echo time: 1.25 ms, flip angle: > 50°, spatial resolution: 1.3 × 1.3 × 6 mm and typical temporal resolution of 30 ms. A stack of images covering the left ventricle in the short axis as well as images in the long axis views were acquired. In addition, phase contrast velocity mapping was acquired in the main pulmonary artery to quantify pulmonary regurgitation.

### Image analysis

Images obtained from CMR were analysed using the software Segment (http://segment.heiberg.se) [13]. Ventricular volumes were measured in short-axis views by delineation of the epicardial and endocardial contours of the respective ventricles. End-systole was set to the timeframe just before opening of the atrioventricular valves with the lowest intraventricular volumes and end-diastole to just before start of ventricular contraction where the intraventricular volume was the greatest. Stroke volume was calculated as end-diastolic volume-end-systolic volume. Ejection fraction was computed as (SV/EDV) × 100%. For PR fraction, when values were obtained in both breath hold and free breathing, a mean was calculated. All volumes were indexed to body surface area (BSA) to account for variations in body size. Global longitudinal RV strain was measured using feature tracking in 4 chamber cine CMR images, encompassing both the free wall and the septum. The RV was manually delineated, with semi-automated myocardial tracking propagated throughout the cardiac cycle. Manual adjustment was applied if necessary. TAPSE (tricuspid anular plane systolic excursion) was measured by semi-automatic tracing of the insertion point of the RV free wall.

### Atrioventricular plane displacement and quantification of longitudinal contribution to stroke volume

Tracking of atrioventricular plane displacement [10] was semiautomatically conducted in Segment using a validated algorithm [14]. In one patient the CMR image did not include a four-chamber view and this patient was therefore excluded from the analysis.

**Fig. 1:**
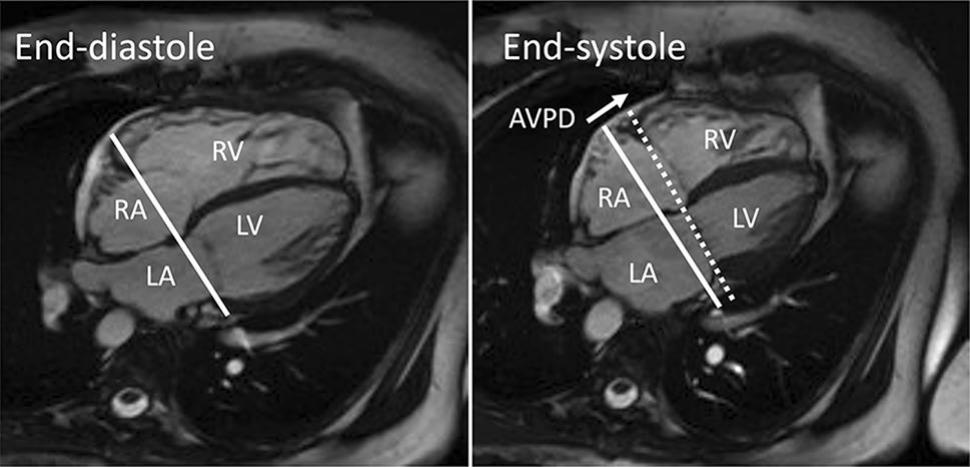
Measurements of atrioventricular-plane displacement (AVPD). APVD is the distance of the atrioventricular plane movement from diastole (solid line) to systole (dashed line). *RA* right atrium, *LA* left atrium, *RV* right ventricle, *LV* left ventricle

Longitudinal contribution to right and left ventricular stroke volume, respectively, was calculated in CMR images as the AVPD distance multiplied by the mean ventricular epicardial volume in the slices encompassed by the systolic excursion of the AV-plane [10, 11, 14] as seen in Fig. 1. Longitudinal contribution is presented as percentage of the planimetric SV for each ventricle.

### Quantification of septal movement and volume

The ventricular septum was defined by the RV insertion to the LV in ED and ES. Septal volume was defined as the volume encompassed by septal movement between the RV insertion points, from ED to ES, globally. Note that septal volume was not defined as the largest difference in volume but rather from ED to ES, thus minimizing impact of potential asynchronous contraction of the septum. At the base of the heart and the apex, only images where the ventricular septum was present in both ED and ES were used. Septal volume is presented in millilitres where positive values indicate movement towards the left side of the heart and negative values indicate movement towards the right side of the heart. The latter would indicate a positive contribution to RVSV. To investigate the effect of asynchronous septal contraction due to the presence of right bundle branch block septal contribution to stroke volume was calculated for each slice encompassing septal insertion points as above. Additionally the correlation between septal contribution to stroke volume and QRS-duration is illustrated.

### Statistical analysis

GraphPad Prism 8.4.2 for Mac (Graphpad Software San Diego, CA, USA) was used for statistical analyses. Descriptive statistics and continuous variables are presented as median [interquartile range]. To determine interventricular relationships, paired Wilcoxon test (non-parametric) was used. Association between variables were analysed by Pearson correlation. A P-value of < 0.05 was considered statistically significant.

## Results

### Patient characteristics and image acquisition

Table 1 shows patient characteristics. Eighteen patients with surgically corrected Tetralogy of Fallot scheduled for pulmonary valve replacement were retrospectively included in the study. Primary surgery was conducted at median 6 months of age [3–12 months]. All patients had right bundle branch block on ECG.

**Table 1:** Patient Characteristics. Values are median [interquartile range]

Patient characteristics	*N*	*Median[Q1-Q3]*
Age at CMR (years)	18	17 [14–28]
Age at primary surgery (months)	15	6 [3–12]
Females n(%)	1 (6)	
Myectomy/Valvotomy	1	
RV-patch	5	
Transannular patch	4	
Unknown/Other	9	
NIBP (mmHg) Systolic		113 [108–115]
NIBP (mmHg) Diastolic		63 [58–67]
Primary surgery to CMR (years)	13	15 [12–21]
CMR to catheterization (months)		13 [4–36]

*RV* right ventricle, *NIBP* non-invasive blood pressure

Cardiac magnetic resonance imaging was performed at age 17 years [14–28 years] with time after initial total correction 15 years [12–21 years] (for 5 patients date of initial correction was unknown). Catheter measurements were obtained between May 2010 and January 2019. CMR acquisition was performed between August 2009 and August 2017.

All patients had PVR performed following CMR. Out of 10 patients who had RVEDVi < 140 ml 8 had pulmonary stenosis (defined either as a pressure drop across the pulmonary valve > 30 mmHg using catheterization or an echocardiographic gradient > 20 mmHg) as their indication for PVR. Two patients qualified for PVR due to a combination of RV dysfunction, pulmonary regurgitation and borderline RVEDVi.

### Ventricular volumes, ejection fraction and pulmonary regurgitation

Table 2 shows CMR-obtained volumes. RV volumes were larger than corresponding LV volume and RVEF was lower than LVEF (P < 0.0001). Two patients had mild regurgitation (PR fraction < 20%), four had moderate regurgitation (PR fraction 20–35%) and 12 showed severe PR regurgitation (PR fraction > 35%)[15].

**Table 2:** Cardiac magnetic resonance measurements. Values are median [interquartile range]

CMR measurements	Median[Q1-Q3]	Median[Q1-Q3]	P-value
CO (L/min)	5.1 [3.5–6.0]		
Cardiac index (L/min/m2)	2.8 [2.5–3.4]		
Septal volume (ml)	− 2 [− 4–3]		
PR regurgitant fraction %	48 [32–57]		
TAPSE (mm)	13 [9–18]		
RV lateral s´ (mm/s)	63 [55–80]		
RV lateral e´(mm/s)	78 [69–88]		
RV lateral a´(mm/s)	68 [47–78]		
	Left ventricle	Right ventricle	
EDV (mL)	152 [130–191]	229 [190–295]	< 0.0001
EDVi (ml/m2)	83 [73–104]	143 [114–185]	< 0.0001
ESV (mL)	75 [58–102]	149 [121–188]	< 0.0001
ESVi (ml/m2)	47 [32–58]	88 [75–114]	< 0.0001
SVi (ml/m2)	41 [35–48]	48 [39–63]	0.26
Long. Cont. SV (%)	54 [47–64]	49 [41–53]	0.039
EF (%)	47 [44–52]	35 [31–39]	< 0.0001

*CO* cardiac output, *PR* pulmonary regurgitation, *TAPSE* tricuspid annular plane systolic excursion, *RV* right ventricle, *EDV* end-diastolic volume, *EDVi* end-diastolic volume indexed, *ESV* end-systolic volume, *ESVi* end-systolic volume indexed, *SVi* stroke volume indexed. Long. Cont. *SV* longitudinal contribution to stroke volume, *EF* ejection fraction

Longitudinal contribution to RVSV was lower (49%, 51–53) than longitudinal contribution to LVSV (54%, 47–64, p = 0.039). Two patients did not contribute with values for TAPSE and one patient did not contribute with longitudinal strain, this due to insufficient CMR coverage (lack of applicable views for each analysis).

**Fig. 2:**
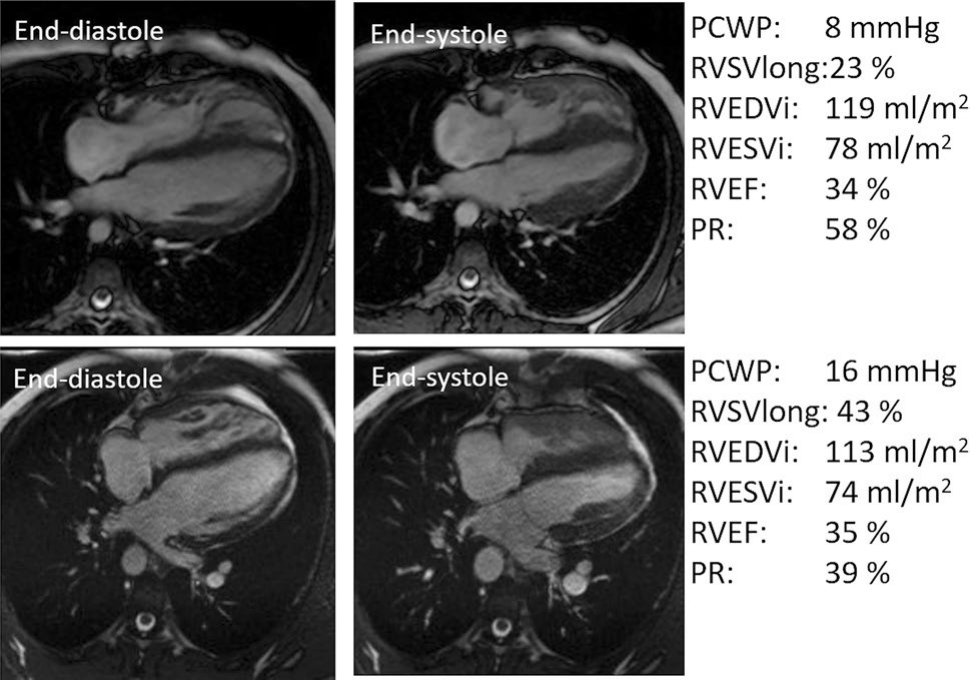
Right ventricular longitudinal contribution to stroke volume (RVSVlong %) is associated with precapillary wedge pressure (PCWP) regardless of change in ventricular volumes, ejection fraction and degree of regurgitation. *RVEDVi* right ventricular end-diastolic volume indexed to BSA, *RVESVi* right ventricular end-systolic volume indexed to BSA, *RVEF* right ventricular ejection fraction, *PR* pulmonary regurgitation fraction

Figure 2 shows CMR images from two different subjects with obtained values for illustrative purposes.

### Invasive measurements

Recordings of PCWP were performed in both the left and right pulmonary artery (PA) (n = 3), left PA only (n = 10) or right PA only (n = 5). Pulmonary artery pressures were recorded in the pulmonary branches (n = 18) and in the main pulmonary artery or the surgically placed conduit (n = 14). Right ventricular pressure was obtained in all patients. Pressure drop across the pulmonary valve was 33 mmHg [16–49 mmHg]. 10 patients were characterized as having pulmonary stenosis (> 30 mmHg pressure drop). The transpulmonary gradient was 9 mmHg [7–11 mmHg], with two patients showing a transpulmonary gradient > 12 mmHg consistent with pulmonary hypertension. See Table 3 for full details.

**Table 3:** Catheter data. Values are median (interquartile range)

Cath data (mmHg)	Median[Q1–Q3]
Left PCWP	11 [8–15]
Right PCWP	11 [10–12]
RV systolic pressure	55 [43–79]
RV end diastolic pressure	10 [7–12]
Pulmonary valve pressure drop	33 [16–49]
Transpulmonary gradient	9 [7–11]

*PCWP* pulmonary capillary wedge pressre, *RV* right ventricle

### Correlations between ventricular function, volume load and left sided filling pressures

**Fig. 3:**
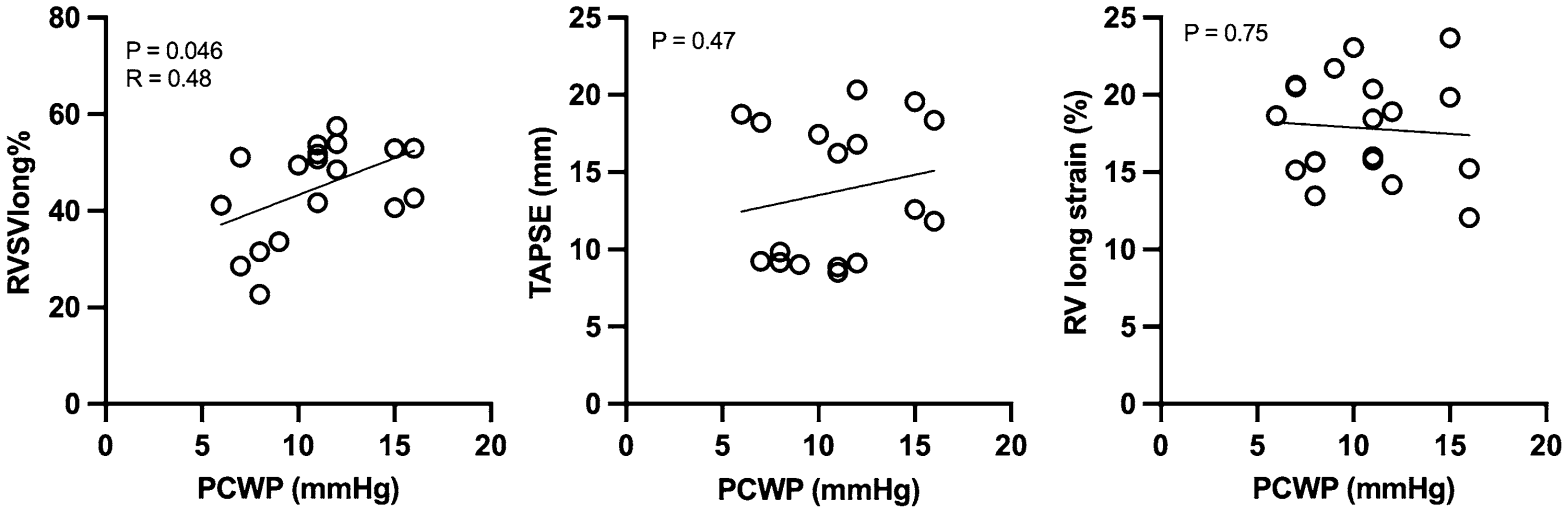
Correlation analysis between measurements of RV longitudinal function and precapillary wedge pressure (PCWP). *RVSVlong* % right ventricular longitudinal contribution to stroke volume, *TAPSE* tricuspid annular plane systolic excursion

**Fig. 4:**
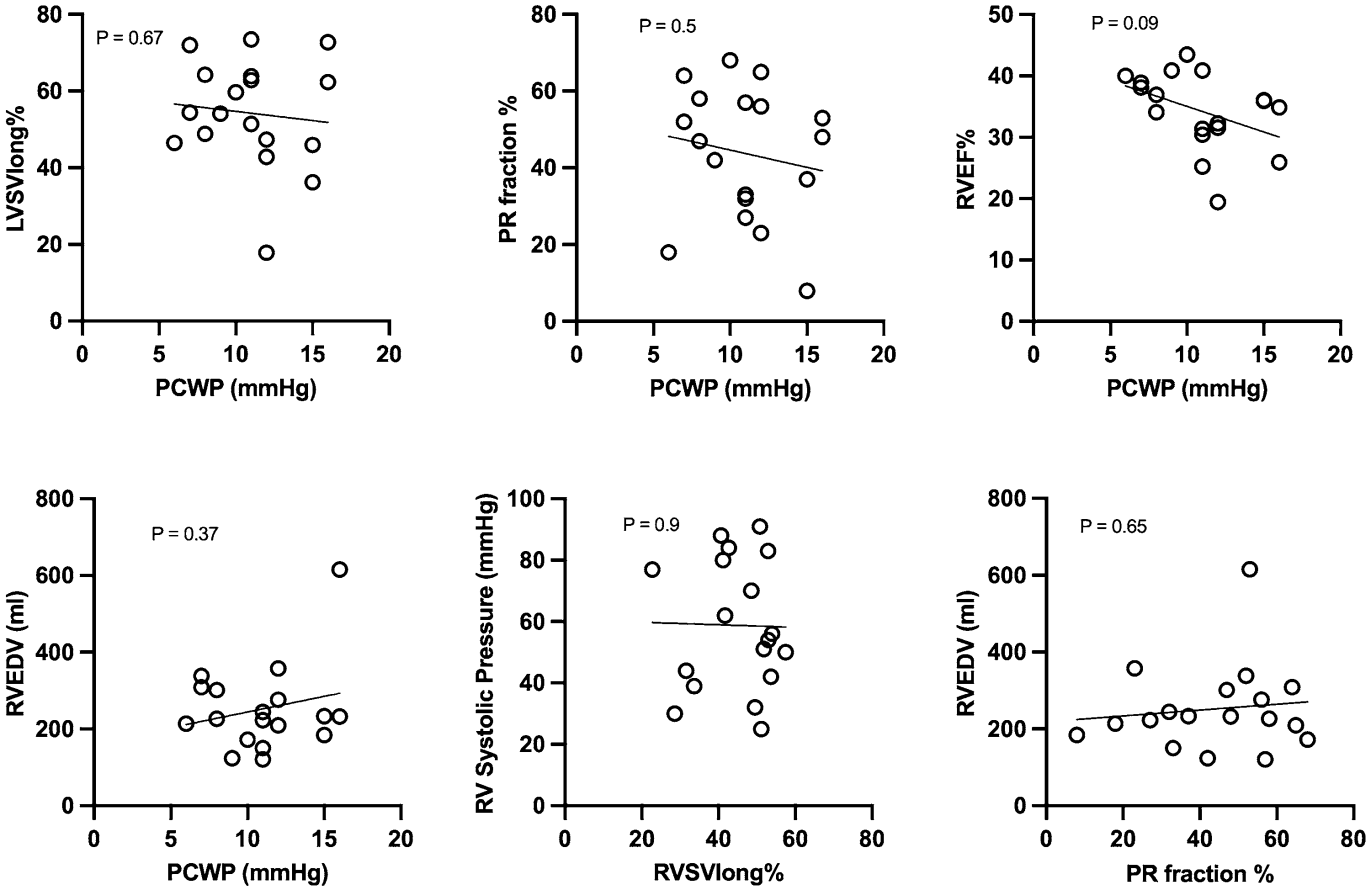
Correlation analysis between LV and RV functional measurements and precapillary wedge pressure (PCWP). *LVSVlong* % left ventricular longitudinal contribution to stroke volume, *RVEF *% right ventricular ejection fraction. *PR* pulmonary regurgitation

A moderate positive correlation was found between longitudinal contribution to RV stroke volume and LV filling pressure measured as PCWP (r = 0.48, p = 0.046) as presented in Fig. 3. This correlation was not present when comparing RV global longitudinal strain with PCWP nor when using TAPSE as measurement of right ventricular longitudinal function as seen in Fig. 3. Right ventricle ejection fraction showed no significant correlation with PCWP, presented in Fig. 4. Right ventricle volume load measured as pulmonary regurgitant fraction did not correlate with left-sided filling pressures (p = 0.5) and neither did RV EDV (p = 0.65, Fig. 4). On the left side of the heart, there was no correlation between longitudinal contribution to stroke volume and filling pressure of the LV.

### Septal contribution to stroke volume

**Fig. 5:**
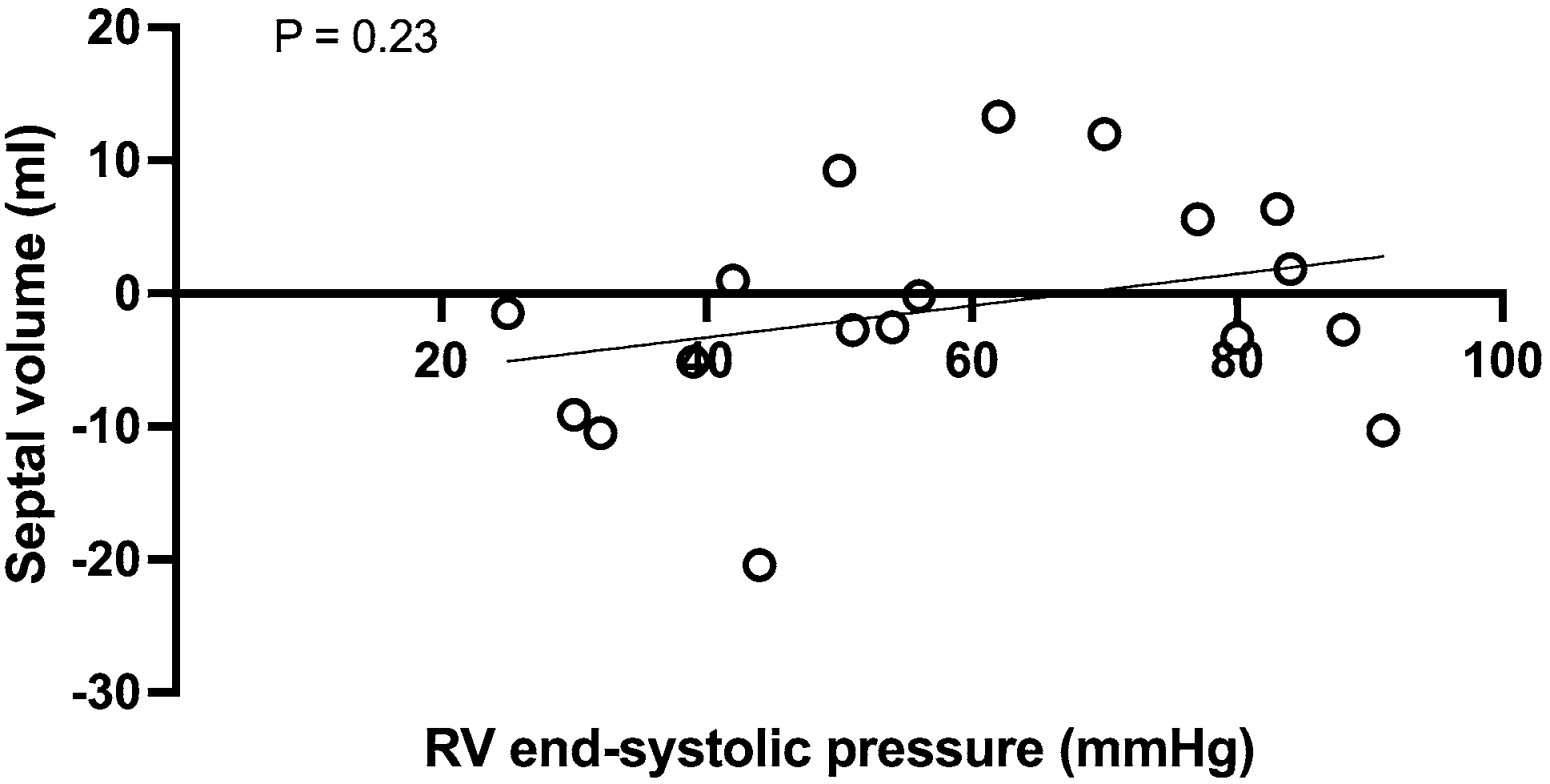
Septal volume in relation to right ventricular end systolic pressure. positive values indicating movement towards the right side of the heart and negative values towards the left side

Septal contribution to SV was − 2 ml [− 3.8–2.1]. Seven patients had positive values indicating movement towards the LV and thus septal contribution to LVSV. Eleven had negative values indicating movement towards the RV and thus septal contribution to RVSV. As illustrated in Fig. 5, septal contribution to SV did not correlate with RV end-systolic pressure. Analysis of septal movement towards the left or right ventricle in all image slices from base to apex were performed and showed no differences in movement in the septum in the basal, midventricular vs apical slices between subjects. Patients with a leftward movement and thus positive contribution to LV SV, in general had this movement throughout the septum.

**Fig. 6:**
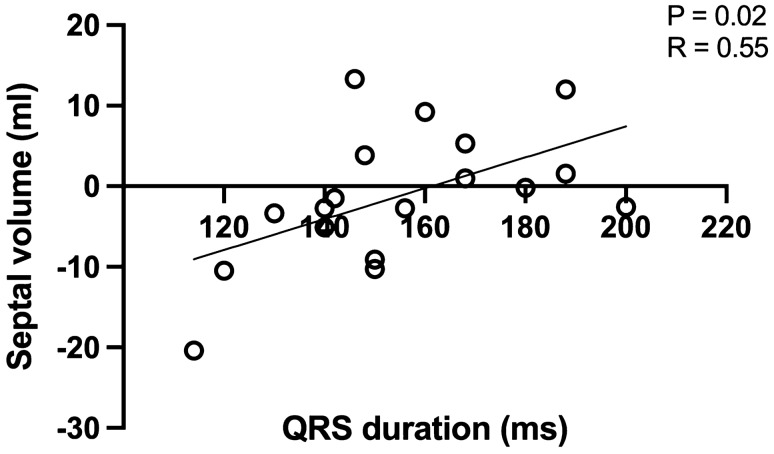
Septal movement and QRS-duration

Septal volume indicating movement towards the LV in systole showed a weak positive correlation with longer QRS-duration (p = 0.02, r = 0.55), illustrated in Fig. 6.

## Discussion

This study addresses the hemodynamic relationship between LV filling pressures (measured as PCWP) and longitudinal RV pumping in patients with rToF. In addition, we investigate the direction of septal movement and its relation to RV end systolic pressure. Right ventricular longitudinal contribution to SV and left side filling pressures showed a moderate positive correlation. These results show that the previous experimental findings of a correlation between RV longitudinal function and LV filling pressure, measured as PCWP, in the setting of PR [12] are applicable in humans with rToF. When using RV global longitudinal strain, RV EF or TAPSE as measurements of RV longitudinal function, correlations with LV filling pressures were not seen. This is in line with previous findings by our group showing that AVPD, TAPSE and strain are not clinically interchangeable and that AVPD might reflect aspects of longitudinal function that TAPSE and strain does not [8]. Right ventricular ejection fraction showed no significant correlation with PCWP. Degree of pulmonary regurgitation and RV end-diastolic volume, as measurements of RV volume load, did not show any significant correlation with PCWP, as seen in Fig. 4. Septal movement was not related to RV end systolic pressure which may be due to the combination of volume and pressure load, the inherent cardiac defect or scarring post initial surgical repair. QRS duration affects septal movement and a tendency towards asynchronous movement was found when comparing direction of septal movement in different parts of the septum.

As long-term survival with rToF is improving, the importance of understanding the hemodynamics of these congenital heart defects become apparent [16] while timing of PVR after initial surgical correction remains debated [17]. A better understanding of the intrinsic relationship between the left and right sides of the heart in rToF and the importance of intracardiac pressures [18] will aid in developing non-invasive measurements and to guide future therapeutic interventions.

We sought to study RV longitudinal function in relation to LV filling as further indices to evaluate biventricular function in rToF is needed. RV dilatation has long been used as a marker of declining ventricular function in rToF, but recent findings suggest that this may be too simplistic. Rashid and colleagues highlighted this and showed that RV systolic function was more important for exercise capacity (an ultimately cardiac-output-dependent variable) than RV dilatation in rToF [19]. TAPSE, strain and AVPD are all modalities to measure ventricular longitudinal function but these have been shown not to be directly interchangeable in patients with rToF [8].

In 2014, Stephensen et al*.* [9] studied the relationship between longitudinal contribution to RVSV and volume load of the RV. In theory; preservation of longitudinal movement of the atrioventricular plane displacement (AVPD) in a volume loaded RV, such as during pulmonary regurgitation, would result in the RVSV exceeding the LVSV due to “overfilling” of the right atrium when the lowering of the atrioventricular plane results in aspiration of blood from the caval veins to the right atrium while at the same time filling the ventricle with a pendulum volume from the pulmonary regurgitation. As such, lower longitudinal contribution to RVSV could be an adaptive pumping mechanism and a lower longitudinal contribution to RVSV have previously been seen in rToF patients [8]. Lower absolute values for longitudinal contribution to RVSV in rToF might thus be of compensatory nature.

Quantification of left sided filling pressure using precapillary wedge pressure is used clinically when a left heart catheterization is not available, but LV end-diastolic pressure (LVEDP) is considered the gold standard measurement of LV filling pressure. Precapillary wedge pressure do underestimate LVEDP, and is affected by pulmonary congestive disease more so than LVEDP [20, 21]. One can thus argue that PCWP is less consistent with LV pressures in the setting of potential pericardial, and myocardial, scarring and possible constrictive properties of the heart post open heart surgery. This need to be taken into account when interpreting results, as discussed by Reddy et al. [22]. The findings in this study supports the hypothesis that longitudinal pumping is of importance for LV filling.

Kopic et al*.* showed that volume load affects longitudinal pumping in an experimental model where isolated PR lead to decreased longitudinal pumping and when pulmonary valve integrity was restored (by pulmonary valve replacement) longitudinal pumping increased once again [12]. The same was true when evaluating rToF-patients after PVR; RV longitudinal function (measured as longitudinal strain), increases immediately after PVR [12], however, with time RV longitudinal function continues to decrease even after alleviating the volume load on the RV [23]. Thus, volume load as a single cause of impaired RV longitudinal function in rToF might be too simplistic. To summarize; RV longitudinal contraction assessed using CMR might be a valuable measurement of ventricular function even after minimizing volume load with PVR.

In this study we found that RV longitudinal contribution to stroke volume is related to PCWP, with greater longitudinal contribution being related to higher filling pressures of the left ventricle. The same correlation was indeed observed by Kopic et. al in a porcine model of isolated PR [12], and one explanation presented was that volume “shortage” on the left side of the heart due to regurgitating volume over a non-patent pulmonary valve would require a greater longitudinal contraction to uphold filling pressures (PCWP). Kopic et. al could undoubtedly show signs of underfilling of the LV with LVSV being lower when pulmonary regurgitation was present, indicating lower effective RVSV (RVSV minus PR volume). This contradicts the notion that lower longitudinal pumping would be favourable in a volume-loaded RV [9] and instead promotes longitudinal pumping as a necessity to uphold left-sided pressures. The stronger correlation between RV longitudinal function and LV filling pressures shown by Kopic et al*.* could be attributed to the use of an animal model rather than a heterogenous patient cohort. No correlation between degree of PR and PCWP could be shown in this study in patients with rToF nor in the above-mentioned experimental study of PR. Instead, PCWP was only related to RV longitudinal function and this suggests that patients with the same size pulmonary regurgitant volume reacts differently with regards to longitudinal function. As mentioned previously the correlation between increased RV longitudinal contribution to SV and increased PCWP could potentially be, at least partly, due to restrictive properties of the left atria and ventricle, thereby putting a greater “demand” on the RV pumping capacity to uphold filling. To what degree scarring and constrictive physiology of the ventricles after open heart surgery results in raised RV longitudinal contraction remains to be studied.

The current results suggests that RV longitudinal function may be of importance for the filling of the left atria and left ventricle, regardless of volume load on the RV. This notion is strengthen in the light of previous studies showing lower left atrial volumes and left ventricular volumes (lower LVEDV) using echocardiography [24, 25] as well as decreased left atrial volumes using CMR [8] in patients with rToF and lower RV longitudinal contraction. Whether this is due the RV not being able to “deliver” adequate volumes (RV dysfunction) or to atrial dysfunction or scarring of the atria or ventricle after opening of the pericardium is yet to be determined.

Paradoxical septal movement towards the right ventricle during systole, was seen in a majority of patients included in this study. Paradoxical septal movement in rToF have previously been shown to be associated with lower LVEF [26] and is a sign of RV volumetric overload [27]. In our study, septal movement was predominantly towards the RV at lower RV end-systolic pressures consistent with the volume load of PR rather than pressure load. When RV end-systolic pressures where higher, septal movement could inconsecutively be both right-bound or left-bound, contributing to either right or left ventricular pumping, as shown in Fig. 5*.* We would therefore conclude that septal movement is a less reliable indicator of ventricular function when there is a combination of volume- and pressure load, such as in the case of combined pulmonary insufficiency and stenosis. Surgical patch closure of a ventricular septal defect in rToF may affect interpretation of septal mechanics, as well as asynchronous contraction of the septum due to right bundle branch block, the latter being illustrated in Fig. 6. No differences in movement in the septum in the basal, midventricular or apical slices. Patients with a leftward movement and thus positive contribution to LV SV, in general had this movement throughout the septum. Variations in septal contribution across slices were small. However, a correlation between duration of the QRS-complex and direction of septal movement was seen and this supports asynchronous contraction of the septum, with QRS-duration having an impact on septal contribution. The lack of correlation between direction of septal movement and measurements of LV filling pressures in combination with variations in contraction suggests that direction of septal motion is an unreliable measurement in patients with rToF.

### Limitations

The cohort studied is a heterogenous one from a tertiary referral centre which may limit generalisability. However, this is often the case when studying seldom occurring congenital heart defects. In this study we did not have sufficiently large patient numbers to conduct subgroup analyses, such as for example for different surgical techniques. This is a major area of discussion as constrictive physiology following open heart surgery may affect intracardiac pressures. Also, both pulmonary stenosis and regurgitation was present in our cohort contributing to the heterogeneity. CMR and catheterization were not carried out simultaneously, therefore we cannot account for changes in hemodynamic properties during the time elapsed between assessments. For example, a continuous deterioration of RV function has been described in rTOF and it can be argued that this might be the case in the studied cohort during the time in between CMR and catheterization and we acknowledge this as a major limitation.

Two patients had pulmonary arterial pressures consistent with definitions of pulmonary hypertension and how concomitant pulmonary vascular pathology might influence the correlation between RV longitudinal function and left sided filling pressures is yet to be determined in this population.

Finally, interobserver variability is an issue when interpreting CMR images for determination of ventricular volumes, this has however been tested and proven robust for the above methods [9].

## Conclusions

Right ventricular longitudinal pumping is associated with left-sided filling pressures in rToF patients but not related to degree of volume overload (RVEF and PR). This is hypothesis generating and suggests that the longitudinal motion of the right ventricle could be viewed as an adaptive mechanism regardless of degree of volume load. Measuring RV longitudinal contribution may be of value when evaluating ventricular dysfunction in rToF-patients. Furthermore, direction of septal movement is an unreliable indicator of RV pressures in rToF patients probably due to combined pulmonary insufficiency and stenosis together with conduction abnormalities following open heart surgery. Care should be taken when interpreting septal movement in the setting of rToF.

## Data Availability

The datasets generated and/or analysed during the current study are not publicly available due to privacy and anonymization but are available from the corresponding author on reasonable request.
