# Correction: Right ventricular longitudinal function is linked to left ventricular filling pressure in patients with repaired tetralogy of Fallot

**DOI:** 10.1007/s10554-023-02854-6

**Published:** 2023-05-22

**Authors:** Martin Johansson, Edem Binka, Benjamin Barnes, Lasya Gaur, Erik Hedström, Shelby Kutty, Marcus Carlsson

**Affiliations:** 1 Department of Clinical Sciences Lund, Clinical Physiology, Lund University, Skåne University Hospital, Lund, Sweden; 2 Department of Clinical Sciences Lund, Pediatric Anesthesia and Intensive Care, Lund University, Skåne University Hospital, Lund, Sweden; 3 Division of Pediatric Cardiology, Johns Hopkins Hospital, Baltimore, MD USA; 4 Department of Pediatrics, Blalock-Taussig-Thomas Heart Center, Johns Hopkins Hospital, Baltimore, MD USA; 5 Department of Clinical Sciences Lund, Diagnostic Radiology, Lund University, Skåne University Hospital, Lund, Sweden

In the original publication the authors have discovered an error in the software algorithm used to calculate radial contribution to stroke volume (SV). The error consists of a failure to account for the slice gap when calculating septal movement and contribution to SV.

Authors correcting note:

Upon reviewing the algorithm used to calculate septal contribution to stroke volume an error was observed. The error consists of a failure to account for the slice gap when calculating septal movement and contribution to SV, affecting images with a slice gap of > 0 mm. After adjusting the algorithm the following changes are to be made in the manuscript:

1. Septal contribution to SV is -3.5 ml (-5.2–4.1) instead of *− 2 ml [− 3.8–2.1]*.
2. Figure 5 has been revised. See revised Fig. 5 attached.
3. Figure 6 has been revised. See revised Fig. 6 attached.

In conclusion; after correctly taking slice gap into account, there is a minor change in the results as described above. However, the main conclusion of the study holds true.

**Fig. 5:**
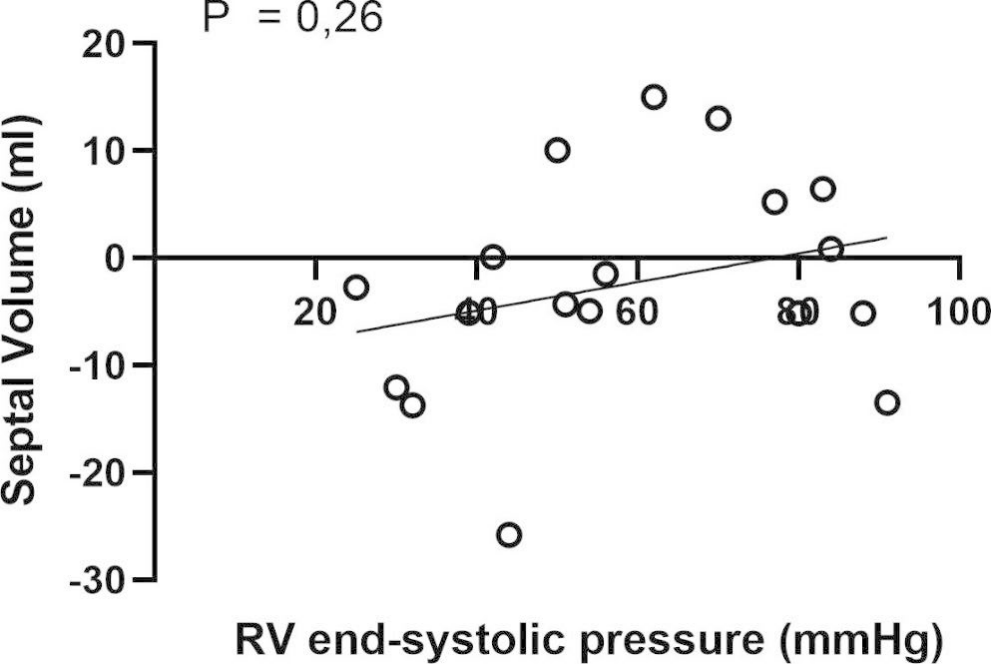
Septal volume in relation to right ventricular end systolic pressure. Positive values indication movement towards the right side of the heart and negative values towards the left side

**Fig. 6:**
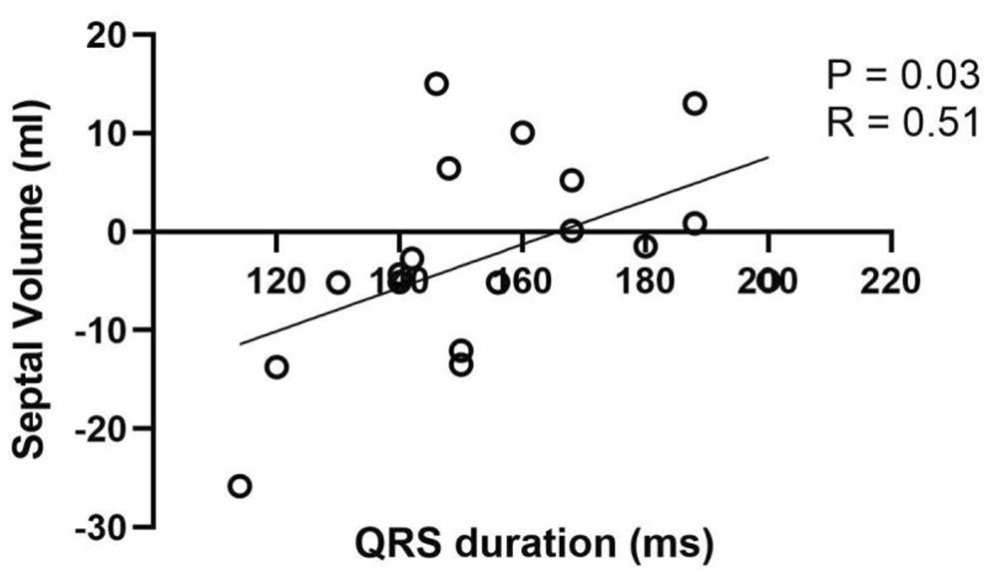
Septal movement and QRS duration

